# Microwave Assisted Pretreatment of *Szarvasi (Agropyron elongatum)* Biomass to Enhance Enzymatic Saccharification and Direct Glucose Production

**DOI:** 10.3389/fpls.2021.767254

**Published:** 2022-01-04

**Authors:** Nicolai D. Jablonowski, Markus Pauly, Murali Dama

**Affiliations:** ^1^Institute of Bio- and Geosciences, IBG-2: Plant Sciences, Forschungszentrum Jülich GmbH, Jülich, Germany; ^2^Bioeconomy Science Center (BioSC), Jülich, Germany; ^3^Institute for Plant Cell Biology and Biotechnology, Heinrich Heine University, Düsseldorf, Germany

**Keywords:** *Szarvasi (Agropyron elongatum)*, microwave pretreatment, lignocellulosic biomass, plant cell wall, biofuels, perennial plants

## Abstract

Biomass from perennial plants can be considered a carbon-neutral renewable resource. The tall wheatgrass hybrid Szarvasi-1 (*Agropyron elongatum*, hereafter referred to as “Szarvasi”) belongs to the perennial Poaceae representing a species, which can grow on marginal soils and produce large amounts of biomass. Several conventional and advanced pretreatment methods have been developed to enhance the saccharification efficiency of plant biomass. Advanced pretreatment methods, such as microwave-assisted pretreatment methods are faster and use less energy compared to conventional pretreatment methods. In this study, we investigated the potential of Szarvasi biomass as a biorefinery feedstock. For this purpose, the lignocellulosic structure of Szarvasi biomass was investigated in detail. In addition, microwave-assisted pretreatments were applied to Szarvasi biomass using different reagents including weak acids and alkali. The produced pulp, hydrolysates, and extracted lignin were quantitatively characterized. In particular, the alkali pretreatment significantly enhanced the saccharification efficiency of the pulp 16-fold compared to untreated biomass of Szarvasi. The acid pretreatment directly converted 25% of the cellulose into glucose without the need of enzymatic digestion. In addition, based on lignin compositional and lignin linkage analysis a lignin chemical model structure present in Szarvasi biomass could be established.

## Introduction

In terms of a sustainable bio-economy, alternative renewable resources have to be identified to replace fossil-based resources. The production of such biogenic resources should ideally avoid land-use conflicts, be ecologically sensible, and should further be easily processable to meet the future demands of raw materials and resulting products in a sustainable manner. Lignocellulosic biomass represents a renewable carbon resource that can be converted into biofuels and other valuable commodity chemicals (Binod et al., 2012; Sasaki et al., 2019). Lignocellulosic biomass consists mainly of the carbohydrate’s cellulose (38–50%) and various hemicelluloses (20–32%), and the polyphenol lignin (10–25%) (Mohammad and Keikhosro, 2008; Pauly and Keegstra, 2008; Kumar et al., 2009). However, the production of lignocellulosic biomass competes with land usage for food/feed production and may have adverse effects on soil and ecological systems (Tilman et al., 2009; Nahm and Morhart, 2018). To address this land-use trilemma for sustainable production of lignocellulosic biomass, certain perennial plants were found to be capable to grow on marginal soils which are unsuitable for food or feed production, and still produce large amounts of biomass. Some prominent examples are e.g., *Sida (Sida hermaphrodita* L. Rusby*), Silphium (Silphium perfoliatum* L.*), Miscanthus (Miscanthus* × *giganteus)*, and *Szarvasi (Agropyron elongatum)* (Csete et al., 2011; Arnoult and Brancourt-Hulmel, 2015; Nabel et al., 2016; Cossel et al., 2020).

The tall wheatgrass cultivar Szarvasi-1, a perennial grass species also known as *Elymus elongatus* subsp. *ponticus* cv. Szarvasi-1 (hereafter referred to as “Szarvasi” only), originated from the Mediterranean basin and has been grown as an energy grass in Hungary to provide biomass for solid biofuel energy production (Csete et al., 2011). The wheatgrass genus is known by various names, represented by *Elymus elongatus* (Host) Runemark (tall wheatgrass), *Agropyron elongatum* (Host) Beauv., *Elytrigia elongata* (Host) Nevski, *E. pontica* (Podp.) Holub, *Elymus varnensis* (Velen.) Runemark, *Lophopyrum elongatum* (Host) A. Löve and *Thinopyrum ponticum* (Podp.) Liu & Wang, among others (Csete et al., 2011). As an intra-specific hybrid of drought-tolerant and robust *E. elongatus* subsp. *ponticus* populations from Hungary and different pontic areas, the Szarvasi-1 energy grass was originally bred in Szarvas (East Hungary), officially recognized by the Hungarian Central Agricultural Office in 2004 (Janowszky and Janowszky, 2007; Csete et al., 2011). While tall wheatgrass has been grown throughout the world for various purposes such as land remediation, erosion control, and forage (Csete et al., 2011), it has recently been proposed as an alternative feedstock crop to maize for biogas production (Dickeduisberg et al., 2017). Szarvasi grass varieties were subsequently adapted in Germany, the Czech Republic, and other countries (Bernas et al., 2019). Szarvasi can grow on semiarid lands and can tolerate a large range of temperatures (35 to –35^°^C). Szarvasi can yield 10–25 tons dry matter/ha biomass per year depending on the soil conditions and water availability (Csete et al., 2011). Recent studies show that Szarvasi biomass yields can be augmented with sewage sludge treatment from communal wastewater (Rev et al., 2017).

Lignocellulosic biomass is recalcitrant toward enzymatic digestion since the polymer’s cellulose, lignin, and hemicellulose are interconnected. For example, the enzymatic accessibility of cellulose is strongly impeded by lignin (Zhao et al., 2021). Biomass pretreatment methods are thus required to open up the lignocellulose to allow enhanced accessibility of enzymes. However, pretreatment processes are the most expensive step in bioethanol production (Agu et al., 2018). Hence efficient pretreatment methods are required to release sugars from lignocellulosic biomass that are then converted by microbes to commodity chemicals. Most pretreatment methods involve a separation step of lignin and hemicelluloses from the biomass to produce a cellulose enriched pulp. The cellulose enriched pulp can then be used as raw material for paper production or further hydrolyzed to glucose. Examples of such pretreatments encompass physical methods such as mechanical comminution (Mayer-Laigle et al., 2018), pyrolysis (Zadeh et al., 2020), physicochemical pretreatments such as steam explosion (Pielhop et al., 2016), ammonia fiber explosion (AFEX) (Mathew et al., 2016), carbon dioxide explosion (Morais et al., 2015), and chemical pretreatments such as ozonolysis (Travaini et al., 2016), acid hydrolysis (Dávila et al., 2021), alkaline hydrolysis (Kim et al., 2016), oxidative delignification (Hernández-Guzmán et al., 2020), an Organosolv Process (Borand and Karaosmanoğlu, 2018), an Organocat method (Grande et al., 2019) and various biological pretreatment methods (Sindhu et al., 2016).

Microwave (MW) pretreatment is one of the suitable pretreatment methods which can be faster and may require less energy compared to conventional methods (Binod et al., 2012). MW treatment involves physical-chemical processes and combines both thermal and non-thermal effects on biomass. Moreover, MW pretreatment disrupts the recalcitrant structures of the biomass by selectively applying the heating on the polar parts in the aqueous environment. While conventional heating transfers the heat through the surface, the microwave produces the heat directly inside the material, which reduces the energy loss in the process. Moreover, the use of MW has advantages in faster energy transfer, controlled heating, shorter residence time, etc. Hence, MW pretreatment can considerably decrease the overall pretreatment time of the biomass and increase the efficiency of the pretreatment (Agu et al., 2018). Several studies have shown that MW pretreatment is an efficient technique to reduce energy consumption during the pretreatment of biomass. However, MW pretreatment conditions such as time, temperature, reagents, and microwave power influence the recalcitrance of the pulp and formation of other by-products (Zhu et al., 2015a,b; Bundhoo, 2018; Sweygers et al., 2018). Combining MW with chemical pretreatments increases the removal of lignin and hemicellulose and increases the accessibility of cellulose to the enzymes of a later saccharification process. Due to these advantages, several microwave pretreatment methods have been optimized for various types of biomass (Bundhoo, 2018). In addition to energy consumption, pretreatment reagents can account for nearly 19% of the total production cost of biofuel production (Kumar et al., 2019). Therefore, the used reagents in the pretreatment process should be economically competitive, act efficiently, and should be recyclable. For example, MW pretreatment of e.g., *Miscanthus* biomass using 0.4 M NaOH or 0.2 M H_2_SO_4_ at 180^°^C shows a higher saccharification efficiency compared to other conditions (Zhu et al., 2015a,b). Another study employing bode wood (*Styrax tonkinensis*) biomass shows that a MW pretreatment with 1.0% (w/w) H_2_SO_4_ produced glucose from cellulose successfully without an enzymatic follow-up step (Sasaki et al., 2019).

Another important prerequisite for an economically competitive bio-refinery process is the valorization of lignin (Ragauskas et al., 2014; Xie et al., 2016). Several chemicals and value-added products can be produced from lignin such as aromatic building blocks, fuels, bulk chemicals, bio-based polymers, antioxidants, immunostimulants, and others (Zakzeski et al., 2010; Sun et al., 2018). However, the lignin composition varies among plant species (HatWeld et al., 2009; Lupoi et al., 2015). For an optimized utilization of lignin in a biorefinery process chain, it is thus important that lignin composition and linkages are structurally and quantitatively characterized (Yelle et al., 2008; Cheng et al., 2013; Lupoi et al., 2015).

In this study, a detailed quantitative analysis of lignocellulosic biomass of Szarvasi is presented including cellulose, hemicellulose, and lignin content and composition using in addition to traditional chemical methodologies also a 2D HSQC (Heteronuclear single quantum coherence) NMR (Nuclear Magnetic Resonance) spectroscopy method. Different microwave pretreatment conditions, using different reagents and concentrations (0.1 M NaOH, 0.2 M NaOH, 0.4 M NaOH, 0.6 M NaOH, 0.1 M H_2_SO_4_, 0.2 M H_2_SO_4_, 0.4 M H_2_SO_4_, 0.6 M H_2_SO_4_, and H_2_O), were applied on Szarvasi biomass. In addition, the production of both pulp and direct glucose conversion were investigated to find suitable microwave pretreatment methods for the Szarvasi biomass.

## Materials and Methods

Plant production and plant material used: Biomass of Szarvasi was produced on fields and was harvested for several years after establishment. The experimental field site was established in May 2012, located in direct proximity to the Research Centre Jülich (Forschungszentrum Jülich GmbH; 50°53′47″ north and 6°25″ n″ east; 80 m a.s.l.) and had a size of approx. 360 m^2^. Seeds of Szarvasi were sown at 32 kg/ha employing a Combiliner Integra 3003, HRB 302 (Kuhn S. A., Saverne, France), and a John Deere 6620 (John Deere, Illinois, United States). The soil type of the field site is a luvisol with a clear gradient of pebble stone share as described elsewhere (Nabel et al., 2017). No fertilizers or pesticides were applied until biomass harvest in its third year of growth, on August 26, 2015. The mean temperature in the year of the harvest was 11.1°C, and the mean precipitation amount accounted for 678 mm. Plants were harvested manually from randomly chosen spots of 1 m^2^. The harvested biomass was dried at 85°C, subsequently milled (<1 mm, Retsch SM 200) and homogenized. Plants of the biomass material used in this study had a median height of 147 cm and a dry matter content of 68%.

Alcohol insoluble residue (AIR) of the biomass was prepared as described elsewhere (Weidener et al., 2020). In brief, Szarvasi plant material (500 mg) was ground to a powder using a PM100 Retsch ball mill in 10 ml grinding jars. The ground material was washed with 70% aqueous ethanol (20 ml) followed by washing the material with methanol:chloroform solution (1:1, v:v, 20 ml a total of 3 times) to obtain the AIR material. The obtained lignocellulosic AIR material was de-starched using α-amylase and Pullulanase (Sigma-Aldrich/Merck KGaA, Darmstadt, Germany). The de-starched AIR material of the Szarvasi biomass was used to quantify the lignin content, lignin composition, crystalline cellulose content, and acetylation content.

Microwave pretreatment procedure: The microwave pretreatment was performed as previously described with some modifications (Zhu et al., 2015b). A sample of 400 mg homogenized Szarvasi biomass was mixed with 16 ml of a pretreatment reagent (different reagents: 0.1 M NaOH, 0.2 M NaOH, 0.4 M NaOH, 0.6 M NaOH, 0.1 M H_2_SO_4_, 0.2 M H_2_SO_4_, 0.4 M H_2_SO_4_, 0.6 M H_2_SO_4_, or H_2_O) in a microwaveable glass reactor (30 ml), which was sealed with a Teflon cap and equipped with a magnetic stir bar. A microwave (Monowave 450) was used to treat the biomass at 180^°^C for 20 min at 600 rpm stirring conditions. After the microwave pretreatment, the mixture was neutralized with 1 M HCl or 1 M NaOH. The neutralized mixture was centrifuged at 4,000 rpm for 20 min to separate the solid biomass (pulp) and hydrolysate fractions. After that, the hydrolysate fraction was separated by careful decantation and stored at –80^°^C for further analysis. The separated solid material (pulp) was rinsed with a small amount of ethanol (5 ml) and dried in an oven at 50^°^C. As mentioned in the literature (Zhu et al., 2015b), the ethanol washing of the solid fraction helps to remove any possible inhibitors formed during the microwave process and also helps to dry the solid biomass fraction quickly.

Structural characterization of Szarvasi AIR: The crystalline cellulose content of Szarvasi biomass and pulp materials were determined as previously described (Foster et al., 2010b). In brief, plant material (2 mg) was treated with 1 ml of Updegraff reagent (Acetic acid:nitric acid:water, 8:1:2 v/v) by incubating 30 min at 100°C. Subsequently, the mixture was cooled to room temperature (RT), the supernatant was discarded, and the pellet dried at 40°C using a nitrogen sample concentrator. The dried pellet was treated with 72% aqueous sulfuric acid (seaman hydrolysis) (Saeman et al., 1954). The resulting hydrolyzed glucose was then treated with the anthrone reagent (2 mg anthrone/1 ml sulfuric acid). The absorbance (625 nm) was measured using a plate reader (Spectra Max, Molecular Devices, LLC. San Jose, CA, United States). Glucose (and hence the crystalline cellulose content), was calculated from the measured absorbance and by establishing the standard curve.

The lignin content of the raw biomass and pulp materials was determined by the acetyl bromide (AcBr) soluble lignin method as described elsewhere (Foster et al., 2010a). In brief, Szarvasi biomass or pulp (1 mg) was treated with freshly made acetyl bromide solution (25% v/v acetyl bromide in glacial acetic acid) at 50°C for 3 h. After neutralization with 2 M NaOH and hydroxylamine hydrochloride, the lignin content was determined by measuring the absorbance at 280 nm using a plate reader (Spectramax instrument, Molecular Devices, LLC. San Jose, CA, United States). Kraft-Lignin (0.1–0.6 mg) was used as a standard (Sigma-Aldrich/Merck KGaA, Darmstadt, Germany).

Lignin composition was determined by the thioacidolysis derivatization method followed by separation of the derivatives using a gas chromatograph coupled with a quadrupole mass spectrometer (GC-MS) as described previously (Foster et al., 2010a). In brief, 200 ml of thioacidolysis reagent [2.5% boron trifluoride diethyl etherate (BF_3_), 10% ethanethiol (EtSH) in dioxane] was added to the Szarvasi biomass or pulp (2 mg) and replaced the remaining air in the sample vial with nitrogen gas. The samples were incubated at 100°C for 4 h and subsequently cooled to RT and, neutralized with 0.4 M sodium bicarbonate. In the following, the dissolved lignin was extracted by ethyl acetate. The extracted lignin was derivatized by adding 100 μl of TMS [N,O-bis(trimethylsilyl) acetamide], 20 μl of pyridine, and 500 μl of ethyl acetate, and the mixture was incubated for 2 h at RT. The lignin composition was determined by using GC-MS (Gas chromatography–Mass spectrometry, Agilent Technologies, Santa Clara, CA, United States) utilizing an SLB-5 column (30 m × 250 μm i.d., 0.25 μm; Sigma-Aldrich/Merck KGaA, Darmstadt, Germany). The employed MS detection settings were: Single-Ion monitoring (SIM) m/z 239, 269, and 299.

The hemicellulosic monosaccharide composition was determined as described previously (Foster et al., 2010b). In brief, 1 mg of dried raw biomass, pulp, or hydrolysates were treated with 2 M trifluoroacetic acid (TFA hydrolysis) for 90 min at 120°C. After incubation, the TFA was evaporated under nitrogen gas at 40°C. The dried sample was dissolved in Ribose solution (internal standard Ribose (30 μg/ml) in water). The released monosaccharides were quantified using an ion chromatography method (IC/HPAEC-PAD, KNAUER Wissenschaftliche Geräte GmbH, Berlin, Germany).

The acetate content was determined as described in an earlier study (Schultink et al., 2015). In brief, Szarvasi biomass and pulp (2 mg) samples were saponified through incubation of 1 M NaOH for 1 h at room temperature. An acetic acid kit (Megazyme Bray, Ireland) was used to detect the acetic acid content and follow the described procedure (Schultink et al., 2015; Megazyme, 2018). The absorbance (340 nm) of the samples was measured using a plate reader (Spectramax instrument, Molecular Devices, LLC. San Jose, CA, United States). The released acetic acid in the supernatant was quantified from the measured values using the formula mentioned in the above literature.

Furfural quantification in the aqueous hydrolysate was achieved by using a published procedure (Martinez et al., 2000). The aqueous hydrolysate was measured using a *BioTek* Power Wave HT UV-VIS Spectrometer at 284 nm.

2D HSQC NMR measurements and quantification: Homogenized Szarvasi biomass AIR and Szarvasi pulp material were further ground in a PM 100 Retsch ball mill as previously described (Cheng et al., 2013). The ground material (25 mg) was dissolved in 0.75 ml of DMSO-d6 containing 10 μl EMIM(OAc) and stirred for 2 h at 600 rpm at 60°C. The dissolved material was transferred to an NMR tube, and a 2D HSQC (Heteronuclear Single Quantum Coherence) NMR spectrum was recorded on a 600 MHz Bruker NMR spectrometer using the following parameters: Pulse program: hsqcetgpsisp.2, NS: 300. The NMR spectroscopic data were processed using Bruker’s Topspin 3.6 software. The chemical shifts were referenced to the solvent DMSO-d6 peak (δ_C_ 39.5 ppm, δ_H_ 2.49 ppm). The degree of xylan *O*-acetylation was quantified as described in Schultink et al. (2015); monosaccharide composition, polymer composition, lignin composition, and lignin linkages were determined as earlier described (Cheng et al., 2013; Grande et al., 2019).

The saccharification (enzymatic hydrolysis) assay was performed as previously published (Damm et al., 2017). The pulp materials (1 mg) were suspended in 0.1 M citrate buffer (pH 4.5). Celluclast enzyme (0.5 μl) was added (from *Trichoderma reesei* (≥700 units/g), Sigma-Aldrich/Merck KGaA, Darmstadt, Germany) and incubated at 50°C for various periods (24–72 h). After enzymatic treatment, the glucose concentration of the samples was determined using a YSI instrument (Hodgson-Kratky et al., 2019).

## Results and Discussion

**FIGURE 1:**
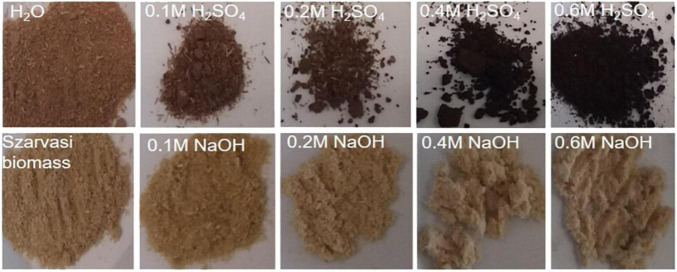
Szarvasi biomass and pulps after various microwave pretreatments including alkaline (NaOH), near neutral (H_2_O), or acidic pretreatments (H_2_SO_4_).

Szarvasi straw was harvested from the field, dried, and de-starched alcohol insoluble residue (AIR) was prepared (Szarvasi biomass) to determine its lignocellulosic attributes (Table 1 and Figure 1). Based on the analysis, Szarvasi biomass represents a typical grass lignocellulosic material such as *Miscanthus* straw or maize stover (Damm et al., 2017). In particular, Szarvasi biomass contains a high amount of xylose content and a significant amount of arabinose in its hemicellulose. Guaiacyl (G)-lignin (52.8%) and significant amounts of *p*-hydroxy phenyl (H)-lignin (3.3%) units (Table 1), which are indicative of a typical grass cell wall (Loqué et al., 2015) were found in Szarvasi biomass as well as Syringyl (S)-lignin (43.9%), which is lower compared to G-lignin (Table 1).

**TABLE 1:** Characterization of szarvasi biomass and pulps after various microwave treatments including alkaline (NaOH), neutral (H_2_O), or acidic pretreatments (H_2_SO_4_).

Pretreatment (Pulp) (± St. dev)	Pulp weight (mg)	Lignin (%)	Crystalline cellulose (%)	Syringyl (S) (%)	Guaiacyl (G) (%)	*p*-Hydroxy-phenyl (H) (%)	S/G ratio	*O*-Acetylation (%)
Szarvasi biomass	400	19.6 ± 0.3^df^	39.7 ± 4.6^d^	43.9 ± 1.1^b^	52.8 ± 2.0^b^	3.3 ± 0.9	0.8	2.4 ± 0.1^c^
0.1 M NaOH	213 ± 7	7.7 ± 0.5^a^	57.5 ± 7.3^a^	44.6 ± 0.1^ab^	53.9 ± 0.3^ab^	1.5 ± 0.2	0.8	0.0^a^
0.2 M NaOH	191 ± 3	4.5 ± 0.7^c^	64.0 ± 2.3^abc^	42.4 ± 0.5^bd^	55.1 ± 0.3^bd^	2.5 ± 0.2	0.8	0.0^a^
0.4 M NaOH	161 ± 6	2.8 ± 1.2^c^	69.4 ± 3.8^bc^	39.8 ± 1.5^cd^	56.0 ± 1.4^cd^	4.2 ± 0.2	0.7	0.02^a^
0.6 M NaOH	146 ± 5	2.3 ± 0.2^c^	72.2 ± 5.8^c^	38.2^c^ ± 0.4	57.0^c^ ± 0.4.	4.8 ± 0.2	0.7	0.0^a^
H_2_O	252 ± 6	17.6 ± 1.3^f^	54.4 ± 4.6^a^	43.7 ± 2.8^b^	53.9 ± 2.6^b^	2.4 ± 0.2	0.8	1.9 ± 0.1^d^
0.1 M H_2_SO_4_	153 ± 17	11.0 ± 1.1^b^	76.0 ± 4.0^c^	40.4 ± 0.1^cd^	57.5 ± 0.0^cd^	2.1 ± 0.2	0.7	0.03^a^
0.2 M H_2_SO_4_	102 ± 4	13.2 ± 0.6^b^	56.6 ± 3.3^a^	47.5 ± 0.2^a^	50.6 ± 0.1^a^	1.9 ± 0.1	0.9	0.06 ± 0.1^a^
0.4 M H_2_SO_4_	68 ± 3	20.4 ± 0.3^d^	28.4 ± 0.3^d^	52.0 ± 0.9^e^	44.9 ± 0.9^e^	3.1 ± 0.5	1.2	0.2^ab^
0.6 M H_2_SO_4_	67 ± 6	25.4 ± 1.1^e^	2.8 ± 3.4^e^	53.4 ± 0.0^e^	42.8 ± 0.4^e^	3.8 ± 0.3	1.2	0.3^b^

*St. dev: standard deviation, n = 3. The superscript letters indicate significant differences based on an ANOVA Tukey HSD test.*

**FIGURE 2:**
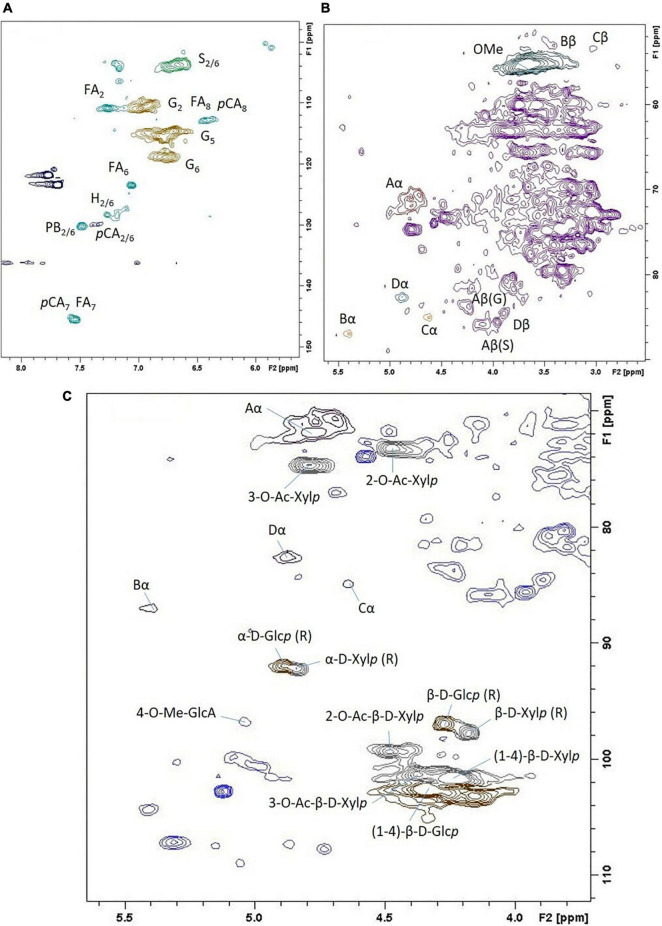
2D ^13^C-^1^H HSQC NMR spectrum of Szarvasi biomass. **(A)** Expansion of the aromatic region of the spectrum; **(B)** aliphatic region; **(C)** anomeric and O-acetylation region. The assignment of the cross-peaks is indicated (for abbreviations see Supplementary Table 1).

**FIGURE 3:**
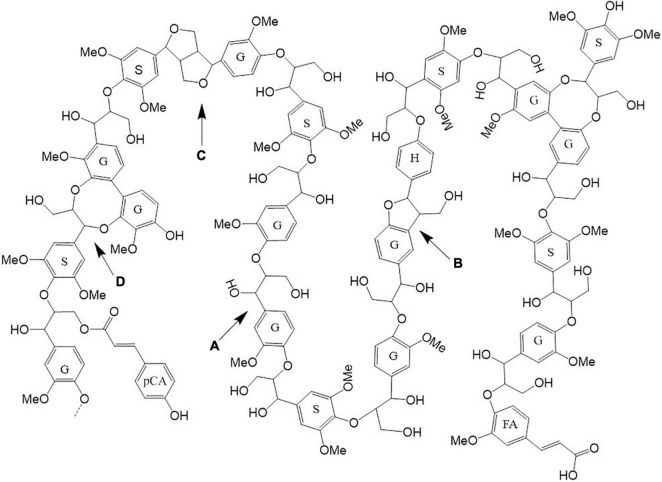
Lignin model structure present in Szarvasi biomass. The lignin model was constructed based on the HSQC NMR data analysis (Supplementary Table 1). Lignin monomers: S, syringyl; G, guaiacyl; H, p-hydroxyphenyl; FA, ferulate; *p*CA, *p*-coumarate. Lignin linkages: A, β-aryl ether; B, phenylcoumaran; C, resinol; D, dibenzodioxocin linkages.

2D HSQC NMR spectroscopy was used to quantitatively characterize Szarvasi biomass in more detail. Such an analysis allows for additional lignocellulosic structural information such as *p*-coumarate and ferulate content and the various linkages between the lignin units (Figure 2 and Supplementary Table 1). This analysis confirmed that Szarvasi biomass represents a typical grass-based biomass due to the presence of *p*-coumarate (*p*CA; 6%) and ferulic acid (FA, 5%; Table 2) and the high abundance of β-aryl-ether (β-O-4) lignin linkages (84%). In addition, Szarvasi biomass also contains other types of lignin linkages such as Phenyl-coumaran (β-5 (Bα), 3.3%), Resinol (β-β (Cα), 1.6%) and Dibenzodioxocin (5-5′/4-O-β′ (Dα), 10.7%), which are typical for grass lignocellulosics. Based on the quantitative data of the lignin analysis a representative model of lignin in Szarvasi could be assembled (Figure 3). This model is very similar to that proposed for another grass straw, namely *Miscanthus* (Cheng et al., 2013).

**TABLE 2:** Release of glucose [μg glucose/mg (biomass/pulp)] by enzymatic hydrolysis at different incubation times and enzyme concentrations of Szarvasi materials (biomass, remaining pulps after microwave pretreatment with various concentrations of acidic or alkali catalyst).

Pulp/biomass μg (glucose)/mg (biomass/pulp) (± St. dev)	Amount of glucose released at different time intervals (hours) and (amount of enzyme used)				
	24 h (0.5 μl)	48 h (0.5 μl)	72 h (0.5 μl)	24 h (5 μl)	48 h (5 μl)
Szarvasi biomass	31.7 ± 3^e^	43.8 ± 3	42.5 ± 5	84.4 ± 6	86.2 ± 5
0.6 M H_2_SO_4_	39.7 ± 4^e^	67.5 ± 5	74.9 ± 8	95.7 ± 7	107.3 ± 9
0.4 M H_2_SO_4_	63.7 ± 13^be^	105.8 ± 26	122.5 ± 34	152.0 ± 26	193.5 ± 33
0.2 M H_2_SO_4_	114.4 ± 16^bc^	188.3 ± 31	212.4 ± 42	335.5 ± 9	468.4 ± 31
0.1 M H_2_SO_4_	164.5 ± 44^cg^	267.4 ± 59	289.3 ± 75	412.5 ± 82	510.5 ± 94
H_2_O	123.8 ± 5^bc^	204.6 ± 7	228.6 ± 6	261.6 ± 12	306.7 ± 17
Crystalline cellulose	275.4 ± 29^f^	442.4 ± 45	494.9 ± 48	627.7 ± 6	782.0 ± 13
0.1 M NaOH	440.2 ± 28^a^	598.5 ± 34	608.7 ± 32	708.4 ± 32	737.5 ± 43
0.2M NaOH	470.6 ± 41^ad^	678.1 ± 24	677.3 ± 22	738.3 ± 64	763.7 ± 54
0.4M NaOH	501.9 ± 32^ad^	686.9 ± 28	690.9 ± 25	791.7 ± 70	820.5 ± 71
0.6M NaOH	529.0 ± 15^d^	729.8 ± 18	733.5 ± 13	836.6 ± 49	870.0 ± 52
Organocat	225.3 ± 42^fg^	N.D	N.D	N.D	N.D

*St. dev: standard deviation, n = 3. Different letters above bars indicate significant differences, assigned based on the ANOVA Tukey HSD test (n = 3). N.D., not detected.*

Szarvasi biomass was subjected to various microwave pretreatments. These pretreatments included ultrapure water (pH 5.5), various acid (0.1 –0.6 M H_2_SO_4_), and various alkali (0.1–0.6 M NaOH) concentrations. After the various microwave pretreatments, the remaining solids (pulps) were separated from the hydrolysate by centrifugation and both fractions were analyzed further.

Depending on the treatment, various colorizations of the pulps could be observed (Figure 1). The pulps after NaOH pretreatments appeared more bleached with higher alkali concentrations, whereas the pulp of the H_2_SO_4_ pretreatments turned dark brown with higher acid concentrations (Figure 1). The darker color might be due to the precipitation of cleaved lignin onto the cellulose surface of the pulp (Li et al., 2007; Zhu et al., 2015b). Lignin precipitation on the surface of the pulp fibers was also observed in ethanol-based Organosolv processing of wheat straw by studying the pulp material through a scanning electron microscope (Xu et al., 2007).

As shown in Table 1 the microwave pretreatments resulted in significant hydrolysis of components from the Szarvasi biomass as indicated by the weights of the remaining pulps. The microwave treatment in water resulted already in a 37% mass loss (400 mg raw material–remaining pulp 252 mg, Table 1). The addition of an alkaline catalyst resulted in a higher mass loss—at the highest NaOH concentration used (0.6 M) only 36.5% pulp remained. An even more pronounced pulp mass loss was achieved with the acid catalyst—at the highest sulfuric acid concentration, only 17% solids remained. Alkaline pretreatment (10% NaOH, 90°C, 1 h) of Napier grass yielded around 47.4% solid recovery, which is higher compared to 0.6 M NaOH MW pretreatment. However, the Alkaline pretreatment could not efficiently remove the lignin and still contains 19.5% lignin of a total 25% lignin content (Tsai et al., 2018).

The higher concentrations of alkali catalyst lead to a near loss of lignin in the remaining pulp. Considering that the Szarvasi biomass contained 78.4 mg lignin (corresponding to 19.6% in a total of 400 mg, Table 1) the remaining pulp after microwave treatment in the presence of 0.6 M NaOH contained only 3.3 mg (corresponding to 2.3% in a total of 146 mg). This result demonstrates that under these conditions 96% of the biomass lignin was hydrolyzed. The addition of an acid catalyst was not as effective. The lowest concentration of sulfuric acid (0.1 M) leads to a reduction of the initial 78.4 mg lignin before the pretreatment to 16.8 mg after pretreatment (pulp: 11.0% of 153 mg). Increasing the amount of acid catalyst did not decrease the remaining pulp lignin any further. In our study, we obtained better results with 0.6 M NaOH compared to the 0.4 M NaOH MW pretreatment of *Miscanthus* biomass (Zhu et al., 2015b) and compared to Alkaline pretreatment (10% NaOH, 90°C, 1 h) of Napier grass (Tsai et al., 2018).

The crystalline cellulose and lignin content of the remaining pulps were determined by classical chemical means (Table 1) and by 2D HSQC analyses (Supplementary Table 1). At high concentrations of the acid catalyst, most of the cellulose in the pulp was removed with a relatively high percentage of lignin remaining. A similar trend has been shown previously by microwave pretreatment of *Miscanthus* biomass and using acetic acid as an acid catalyst of aspen wood (Li et al., 2007; Zhu et al., 2015b). Also, as published previously, dilute acid pretreatment of Napier grass did not show any effect on lignin removal and showed slightly higher amounts of lignin compared to raw biomass (Tsai et al., 2018). One explanation is the re-deposition of cleaved lignin on the surface of the material by re-polymerization (Li et al., 2007; Zhu et al., 2015b). In addition, under acidic pretreatment conditions, the formation of pseudo-lignin was observed (Sannigrahi et al., 2011). Increasing the concentration of acid catalyst or pretreatment time also increased lignin content by the formation of pseudo-lignin (Shinde et al., 2018).

In contrast, high alkaline catalyst concentrations removed lignin with a high residual cellulose content. The hemicellulose content was reduced in the pulp utilizing both types of catalysts–the higher the catalyst concentration the more of the hemicellulosic sugars were removed from the pulp (Figure 3 and Supplementary Table 1). In particular, the pentosyl-units xylose and arabinose are removed upon pretreatment with higher amounts of both catalysts indicating that the hemicellulose arabinoxylan is hydrolyzed and/or degraded. The acidic catalyst at a low concentration leads to a significant and at higher concentration complete removal of arabinoxylan. Similar results have been obtained previously with microwave pretreatment of *Miscanthus* biomass (Zhu et al., 2015a,b).

The lignin composition and linkage types in the remaining pulps were established using thioacidolysis (Table 1) and 2D HSQC (Supplementary Table 1) methods. Overall the lignin composition did not show major changes upon microwave pretreatment using either acid or alkali catalysts. The addition of the acid catalyst leads to a change of the S/G (Syringyl/Guaiacyl) ratio with a higher abundance of S-lignin in the remaining pulp. Concerning the lignin linkages, the acid catalyst eliminated the resinol and dibenzodioxacin linkages, and the remaining lignin in the pulp contained only the β-aryl-ether (92%) and phenyl coumaran (8%) linkages. Since the alkali treatment leads to a removal of lignin units no linkages could be detected.

The Szarvasi lignocellulosic biomass contains polysaccharides that are *O*-acetylated, but the degree of *O*-acetylation is lower (2.4%; Table 1) compared to the one reported for *Miscanthus* biomass (∼4%) and corn stover (∼3%) but higher compared to rice straw (∼2%) (Bhatia et al., 2020). Based on 2D-HSQC NMR analysis, the Szarvasi biomass contained 26% of *O*-acetylation on xylan (Supplementary Table 1 and Supplementary Figures 1–6), mainly at the *O*-3 position, *O*-2 position, and to a much lesser extent at both *O*-2 and *O*-3 positions of the xylosyl-residue. Microwave pretreatments with both acid and alkali catalysts significantly reduced the degree of *O*-acetylation. In the case of the alkali pretreatment, even at a low concentration, the (xylan) acetyl esters are completely removed.

**FIGURE 4:**
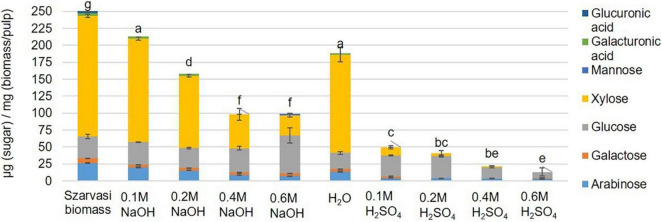
Monosaccharide composition [μg sugar/mg biomass (pulp)] in Szarvasi pulp after different microwave pretreatments. Different letters above bars indicate significant differences, assigned based on the ANOVA Tukey HSD test (*n* = 3).

**FIGURE 5:**
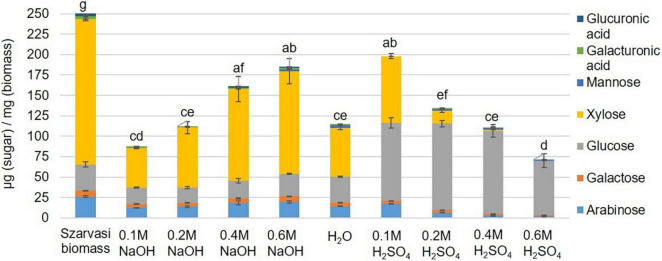
Monosaccharide composition (μg sugar/mg biomass) in Szarvasi hydrolysates of different microwave pretreatments. Different letters above bars indicate significant differences, assigned based on the ANOVA Tukey HSD test (*n* = 3).

The economic competitiveness of processing plant biomass to commodity chemicals demands the utilization of all fractionation streams. Hence, the hydrolysate (liquid phase) of the microwave pretreated Szarvasi biomass was analyzed. Using an acid catalyst in the microwave pretreatment resulted in the release of a large amount of glucose due to the hydrolysis of cellulose (Figure 4). An increase in acid catalyst concentration resulted in a reduction in glucose likely due to the degradation of glucose to furfural, levulinic acid, and hydroxymethylfurfural (HMF) as has been reported in a previous study (Shinde et al., 2018). Indeed, furfural was found in the hydrolysate particularly when higher concentrations of the acidic catalyst were used (Supplementary Table 2). It should be noted that significantly lower amounts of furfural were formed during the microwave pretreatments used here compared to other pretreatment methods (27.5–122.4 mg/L) (Jönsson and Martín, 2016; Grande et al., 2019). In contrast, the use of the alkaline catalyst showed the predominant release of xylose due to the degradation of the hemicellulose arabinoxylan (Figures 4, 5). The conversion of cellulose to glucose was marginal as was the production of furfural. Arabinoxylan was also hydrolyzed using the acid catalyst (xylose content in the pulp, Figure 4). However, little xylose was found in the hydrolysate suggesting that the released xylose was converted into furfural and other chemicals (Aarum et al., 2018; Shinde et al., 2018; Figure 5).

The lignin content in the hydrolysates was quantified as well (Supplementary Figure 7). Adding a catalyst to the microwave pretreatment resulted in an increase of lignin in the hydrolysate in particular when using the highest concentration of the alkaline catalyst. The lignin composition of extracted lignin is different compared to Szarvasi biomass (Table 1 and Supplementary Table 3). The extracted lignin contains a higher abundance of S-lignin and a lower amount of G-lignin compared to the Szarvasi raw biomass. The lignin composition in hydrolysates using higher concentrations of acid catalyst could not be determined with the thioacidolysis method used here, even though the samples contained lignin when using the photometric method (Supplementary Figure 7). One explanation is the formation of condensed, modified lignin sometimes referred to as pseudolignin under these conditions (Aarum et al., 2018; Shinde et al., 2018), which would be detected photometrically, but it’s content of S, G, H-units cannot be determined with the method used here.

**FIGURE 6:**
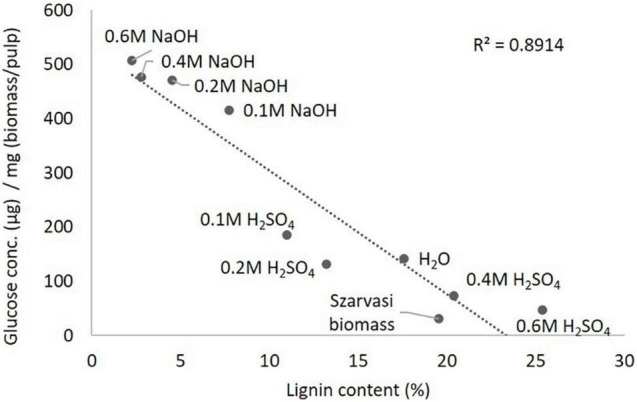
Inverse correlation between lignin content (Table 1) and glucose saccharification yield (Table 2) of various pretreated Szarvasi pulps.

In the current plant biomass conversion process the pretreated pulp material is subjected to enzymatic digestion to convert cellulose to glucose, which can then be fermented by microbes. Hence, the various pulps after the microwave pretreatments were subjected to such an enzymatic saccharification process and the resulting glucose amounts were quantified (Table 2 and Supplementary Figure 8). The saccharification assay results showed that the NaOH pretreatments significantly enhanced the saccharification efficiency of Szarvasi biomass. The saccharification yield of pulp obtained from the 0.1 to 0.6 M NaOH pretreatments are significantly higher compared to untreated Szarvasi biomass and the pulp obtained from H_2_O pretreatment. The addition of the alkaline catalysts enhances significantly the saccharification yield of Szarvasi biomass in particular when using the highest catalyst concentration (0.6 M NaOH). This might be due to a reduction of the cellulose polymer length as well as swelling of the cellulose microfibrils allowing enhanced enzyme access to its substrate (McParland et al., 1982). In contrast to the NaOH pretreatment, the saccharification yield of 0.1–0.6 M H_2_SO_4_ pretreatments was different and the saccharification efficiency of the pulp was decreased with the increase of the concentration of H_2_SO_4_ (Table 2 and Supplementary Figure 8). The addition of the acid catalyst also enhances saccharification yield but to a much lesser extent. Using the highest concentrations of acidic catalyst yields very little glucose during the enzymatic digestion as most of the glucose is already released in the hydrolysate of the pretreatment (Table 2 and Figure 4). The effect of different pretreatments on glucose release in the enzymatic hydrolysis can be clearly observed from Table 2, Figure 6 and Supplementary Figures 8, 9. If a different amount of enzyme was added to each pulp sample based on the crystalline cellulose content, we would expect similar results. In a biorefinery setting enzymes are also supplied to the reaction in excess of substrate and based on biomass rather than substrate, i.e., cellulose, loading.

In addition, at acidic pretreatment conditions, less lignin was removed and/or re-precipitated on the surface of cellulose microfibrils, thus reducing enzyme accessibility to cellulose and removing enzyme due to adhesion (Hu et al., 2012; Li et al., 2018; Santos et al., 2019). Moreover, the formation of lignin-like structures (pseudo-lignins or humins) by degradation of plant polysaccharides during acidic pretreatments (e.g., H_2_SO_4_ or OA) could also prevent the enzyme accessibility to cellulose, which leads to lower saccharification efficiency (Shinde et al., 2018). The saccharification efficiency of the pulp obtained in acid-pretreatment can be improved by removing the residual lignin and by altering the recalcitrance structure of pulp using a post-extraction process with reagents such as phosphoric acid, urea, and ethanol (Huang et al., 2019). Our results show an inverse relationship between lignin content and saccharification yield of the various pretreated pulps, as shown in Figure 6. We observed that even though the 0.1 M H_2_SO_4_ pulp contained higher amounts of cellulose, lower saccharification yields were obtained compared to 0.1 M NaOH pulp, since 0.1 M H_2_SO_4_ contains slightly higher amounts of lignin (11.0%) compared to 0.1 M NaOH pulp (7.7%, Tables 1, 2). Some recent studies showed that lignin can interact with the β-glucosidase or endoglucanase enzymes and could reduce their activities to a very low level by their binding through hydrogen bonding, electrostatic and/or, hydrophobic interactions, or others (Zhao et al., 2021). Moreover, a positive correlation was observed between the cellulose content and saccharification yield of the various pretreated pulps (Supplementary Figure 9). Hence, other acidic catalyst pretreatments such as Organocat yields less glucose in the saccharification assay than the alkaline microwave pretreatment (Table 2).

## Conclusion

A comprehensive analysis of the lignocellulosic structure of the biomass of Szarvasi, a perennial energy grass, demonstrates its similar lignocellulosic attributes compared to other energy grasses. Hence, one can expect similar yields with tested pretreatment and conversion processes carried out on other grass biomass materials.

Under the conditions used here, a microwave pretreatment with an alkaline catalyst (0.6 M NaOH) produced a pulp with significant enrichment in crystalline cellulose and a concomitant low lignin and hemicellulose content. A pulp with such attributes might be advantageous for the production of paper/tissue products. In addition, the enzymatic saccharification efficiency of such a pulp was increased 16-fold when compared to untreated Szarvasi biomass. The pretreatment hydrolysate contained large amounts of monosaccharides such as xylose that can be used for the production of e.g., xylitol, sorbitol, itaconic acid, and/or levulinic acid. Conversely, microwave pretreatments with an acidic catalyst did not result in a reduction in lignin content and hence yielded only slightly higher saccharification yields. However, it should be noted that the use of the acidic catalyst at lower concentrations leads to the direct production of glucose in the hydrolysate of the pretreatment without the necessity of a follow-up enzymatic saccharification step.

## Data Availability Statement

The original contributions presented in the study are included in the article/Supplementary Material, further inquiries can be directed to the corresponding author/s.

## Author Contributions

MD designed the research, performed the experiments, wrote, and edited the manuscript. NDJ performed the plant and agricultural experiments, wrote, and edited the manuscript. MP obtained funding, designed the research, wrote, and edited the manuscript. All authors contributed to the article and approved the submitted version.

## Conflict of Interest

The authors declare that the research was conducted in the absence of any commercial or financial relationships that could be construed as a potential conflict of interest.

## Publisher’s Note

All claims expressed in this article are solely those of the authors and do not necessarily represent those of their affiliated organizations, or those of the publisher, the editors and the reviewers. Any product that may be evaluated in this article, or claim that may be made by its manufacturer, is not guaranteed or endorsed by the publisher.
