# Supplementary material for: Microwave Assisted Pretreatment of Szarvasi (Agropyron elongatum) Biomass to Enhance Enzymatic Saccharification and Direct Glucose Production
Source: Front Plant Sci. 2022 Jan 4;12:767254. doi: 10.3389/fpls.2021.767254 (PMC8765703; doi:10.3389/fpls.2021.767254)
Supplement: Supplementary file 1 [file Data_Sheet_1.docx]

Supplementary Material

# Supplementary Tables

| Structure / (%) | Szarvasi Biomass | Pulp (H_2_O) | Pulp (0.1 M H_2_SO_4_) | Pulp (0.4 M NaOH) | Pulp (0.6 M NaOH) | Lignin (0.2 M NaOH) |
| --- | --- | --- | --- | --- | --- | --- |
| Polysaccharide Composition | |  |  |  |  |  |
| Cellulose | 44.6 | 51 | 73.5 | 73.9 | 79.2 | N.D |
| Hemicellulose | 43 | 39 | 23 | 25.7 | 20.8 | N.D |
| Lignin | 12.4 | 9.9 | 3.5 | 0.3 | N.D | N.D |
|  |  |  |  |  |  |  |
| Monosaccharide Composition | |  |  |  |  |  |
| Glucose | 50.9 | 56.7 | 76.2 | 74.2 | 79.2 | N.D |
| Xylose | 47.8 | 42.5 | 23.3 | 25.7 | 20.8 | N.D |
| Arabinose | 0.6 | 0.2 | 0.5 | 0.1 | 0 | N.D |
| 4-OMe-GlcA | 0.3 | 0.4 | 0 | N.D | N.D | N.D |
| Mannose | 0.4 | 0.2 | 0.1 | N.D | N.D | N.D |
|  |  |  |  |  |  |  |
| Lignin Composition | |  |  |  |  |  |
| Syringal(S) | 31.3 | 34.5 | 39.2 | N.D | N.D | 24.1 |
| Guaiacyl(G) | 62.7 | 59.9 | 58.2 | 100 | N.D | 72.8 |
| p-Hydroxyphenyl | 6 | 5.5 | 2.6 | N.D | N.D | 3 |
| p-Coumarate (*p*CA) | 6.2 | 4.2 | 0 | N.D | N.D | N.D |
| Ferulate (FA) | 5.3 | 6.8 | 0 | N.D | N.D | N.D |
|  |  |  |  |  |  |  |
| Lignin Linkages composition | |  |  |  |  |  |
| β-Aryl ether (Aα) | 84.4 | 71.7 | 91.8 | N.D | N.D | 36 |
| Phenyl Coumaran(Bα) | 3.3 | 8.3 | 8.2 | N.D | N.D | 9.5 |
| Resinol (Cα) | 1.6 | 5.2 | N.D | N.D | N.D | 54.5 |
| Dibenzodioxocin(Dα) | 10.7 | 14.8 | N.D | N.D | N.D | N.D |
|  |  |  |  |  |  |  |
| Acetylation content | |  |  |  |  |  |
| 2-OAc- Xylan | 10.3 | 9.6 | 3.4 | N.D | N.D | N.D |
| 3-OAc-Xylan | 14.5 | 14.8 | 1 | N.D | N.D | N.D |
| 2,3-OAc-Xylan | 1 | 0.9 | 0 | N.D | N.D | N.D |
| Total Ac-Xylan | 25.7 | 25.3 | 4.5 | N.D | N.D | N.D |

Table S1: 2D HSQC NMR analysis of Szarvasi biomass and pulps after various microwave treatments including alkaline (NaOH), near neutral (H_2_O), or acidic pretreatments (H_2_SO_4_ ). N.D. – not detected.

| Hydrolysate | Furfural [mg/L] ± St. Dev |
| --- | --- |
| 0.1M NaOH | 59.8 ± 5.7^ab^ |
| 0.2M NaOH | 57 ± 5.8^ab^ |
| 0.4M NaOH | 62.3 ± 1.0^ab^ |
| 0.6M NaOH | 72.7 ± 5.1^a^ |
| H_2_O | 27.5 ± 1.3^b^ |
| 0.1 M H_2_SO_4_ | 153.8 ± 10.1^cd^ |
| 0.2 M H_2_SO_4_ | 166.7 ± 30.7^c^ |
| 0.4 M H_2_SO_4_ | 173.1 ± 12.5^c^ |
| 0.6 M H_2_SO_4_ | 122.4 ± 4.6^d^ |

Table S2: Furfural content (mg/L) in hydrolysates of different microwave pretreatments. Different letters in superscript indicate significant differences, assigned based on the ANOVA Tukey HSD test.

| Lignin composition in Hydrolysates (%)  (± St. dev) | Syringyl (S) | Guaiacyl (G) | *p-*Hydroxyphenyl (H) |
| --- | --- | --- | --- |
| 0.1 M NaOH | 53.7 ± 0.3^a^ | 45.3 ± 0.3^a^ | 1.1 ± 0.3 |
| 0.2 M NaOH | 51.7 ± 0.7^a^ | 46.1 ± 1.0^a^ | 2.3 ± 1.6 |
| 0.4 M NaOH | 49.3 ± 0.1^ab^ | 48.6 ± 0.3^ab^ | 2.2 ± 0.3 |
| 0.6 M NaOH | 48.5 ± 0.9^ab^ | 49.4 ± 0.2^ab^ | 2.1 ± 1.1 |
| H_2_O | 45.8 ± 0.4^ab^ | 51.2 ± 0.3^ab^ | 3.0 ± 0.1 |
| 0.1 M H_2_SO_4_ | 41.0 ± 10.9^b^ | 49.4 ± 12.7^b^ | 9.6 ± 1.9 |
| 0.2 M H_2_SO_4_ | n.d. | n.d. | n.d. |
| 0.4 M H_2_SO_4_ | n.d. | n.d. | n.d. |
| 0.6 M H_2_SO_4_ | n.d. | n.d. | n.d. |

Table S3: Lignin composition (%) in hydrolysates of different microwave pretreatments. Different letters in superscript indicate significant differences, assigned based on the ANOVA Tukey HSD test.

# Supplementary Figures


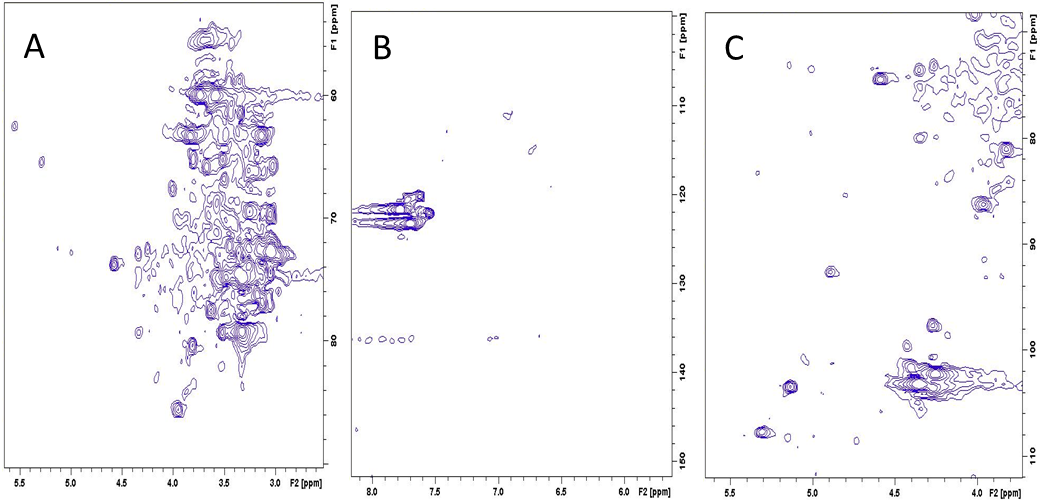


Figure S1: The 2D ^13^C-^1^H HSQC NMR spectrum of Szarvasi Pulp (0.4 M NaOH pretreatment). A. aromatic region, B. aliphatic region and C. anomeric and acetylation regions.


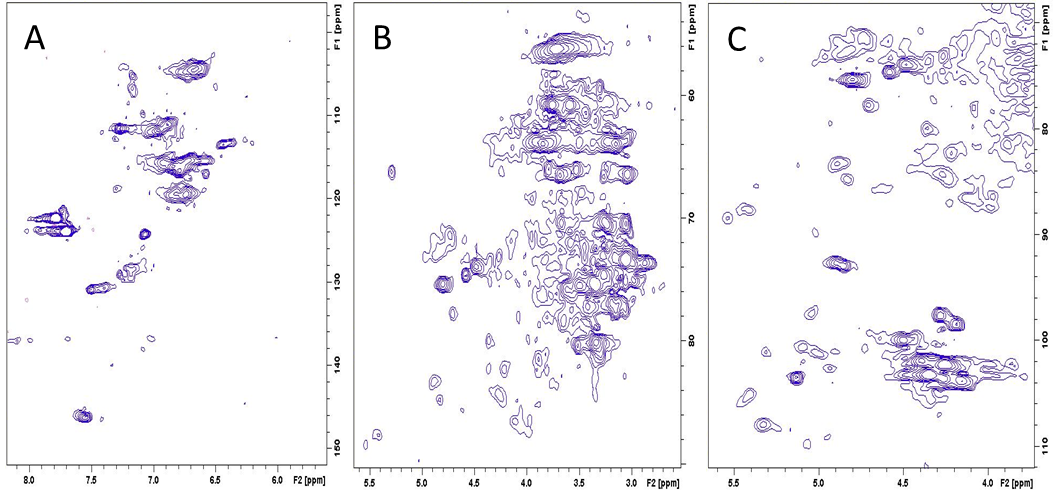


Figure S2: The 2D ^13^C-^1^H HSQC NMR spectrum of Szarvasi Pulp (H_2_O treated). A. aromatic region, B. aliphatic region and C. anomeric and acetylation regions.


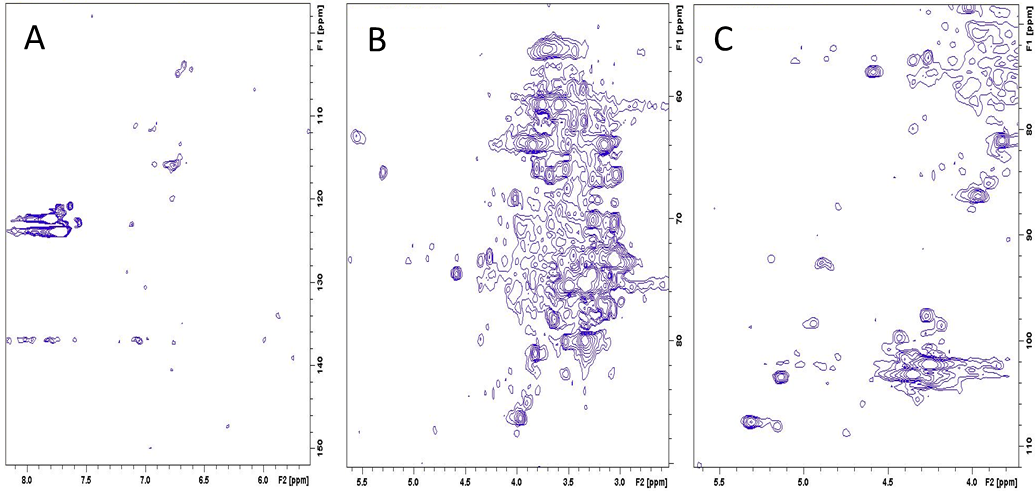


Figure S3: The 2D ^13^C-^1^H HSQC NMR spectrum of Szarvasi Pulp (0.2M NaOH treated). A. aromatic region, B. aliphatic region and C. anomeric and acetylation regions.


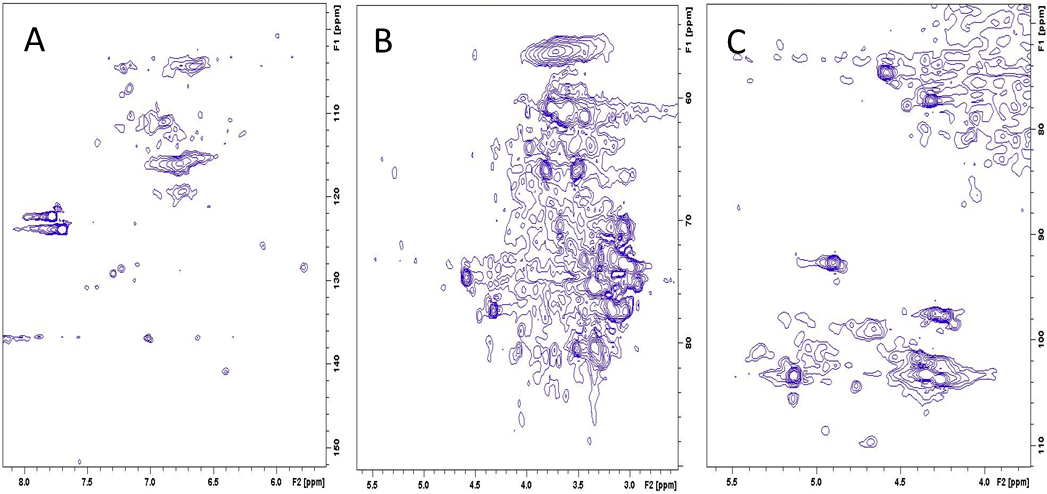


Figure S4: The 2D ^13^C-^1^H HSQC NMR spectrum of Szarvasi Pulp (0.1M H_2_SO_4_ treated). A. aromatic region, B. aliphatic region and C. anomeric and acetylation regions.


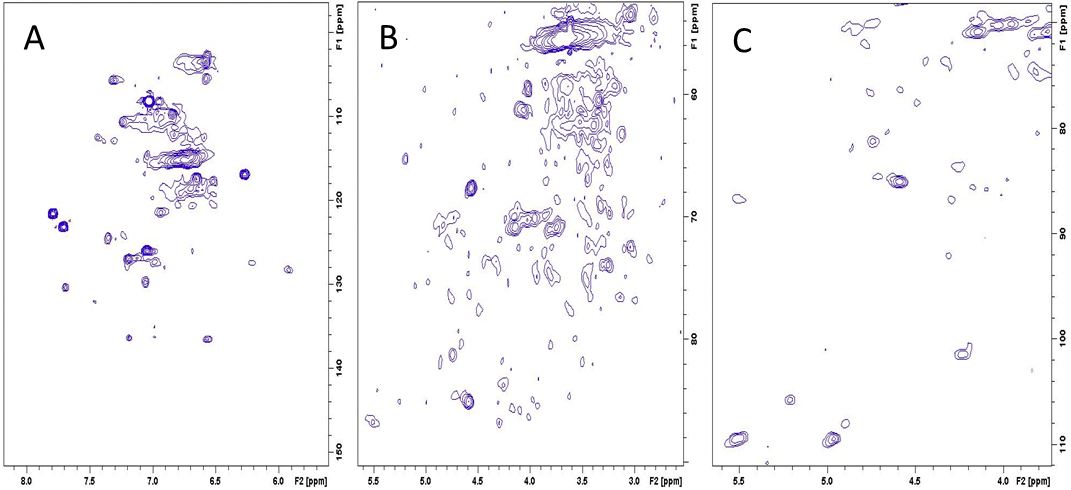


Figure S5: The 2D ^13^C-^1^H HSQC NMR spectrum of Szarvasi extracted lignin (from 0.2M NaOH pretreatment). A. aromatic region, B. aliphatic region and C. anomeric and acetylation regions.


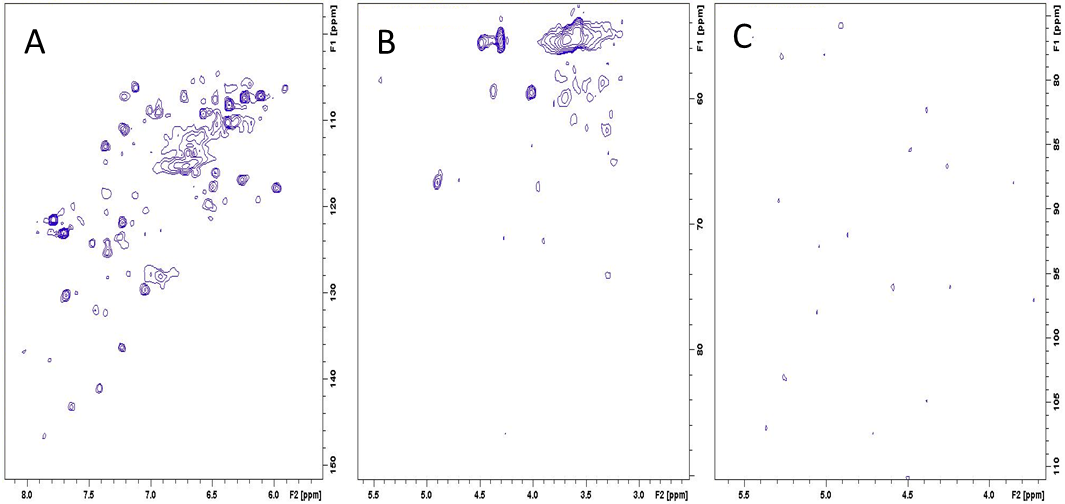


Figure S6: The 2D ^13^C-^1^H HSQC NMR spectrum of Szarvasi extracted lignin (from 0.2M H_2_SO_4_ pretreatment). A. aromatic region, B. aliphatic region and C. anomeric and acetylation regions.


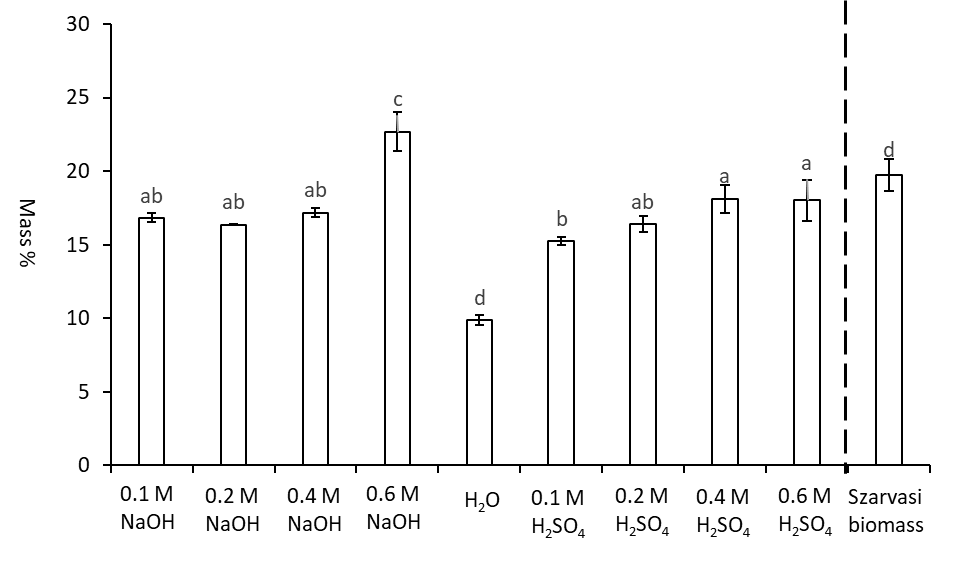


Figure S7: Lignin content (again define the % value in material and methods) in Szarvasi hydrolysates after different microwave pretreatments. Letters were assigned based on the ANOVA Tukey HSD test (n=3).


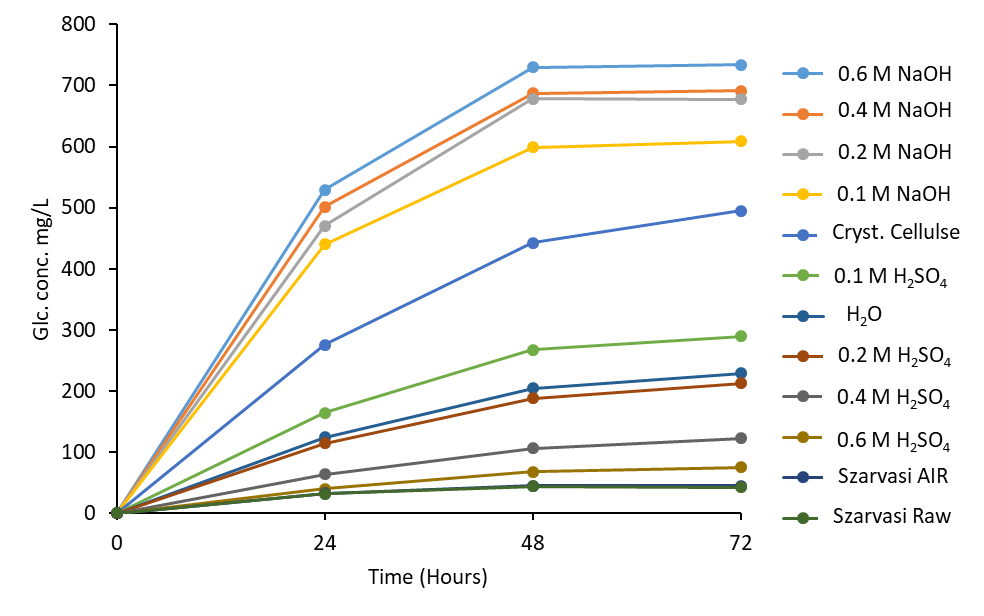


Figure S8: Release of glucose in enzymatic treatment (Saccharification assay) of Szarvasi pulp at different incubation times (24, 48 and 72 hours.


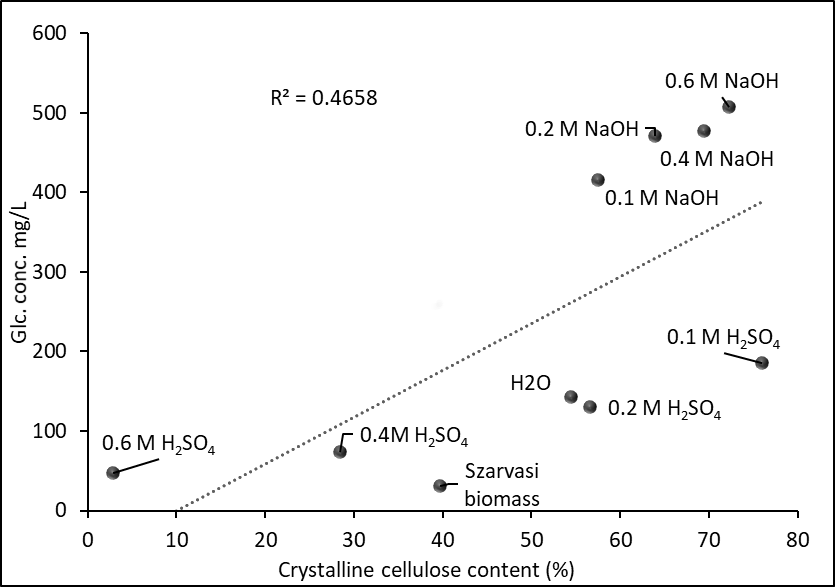


Figure S9: Correlation between crystalline cellulose content and release of glucose (with enzymatic treatment - saccharification) of Szarvasi pulp of different microwave pretreatment conditions.
